# Genome Analysis of Sable Fur Color Links a Lightened Pigmentation Phenotype to a Frameshift Variant in the Tyrosinase-Related Protein 1 Gene

**DOI:** 10.3390/genes12020157

**Published:** 2021-01-25

**Authors:** Andrey D. Manakhov, Maria Y. Mintseva, Tatiana V. Andreeva, Pavel A. Filimonov, Alexey A. Onokhov, Irina E. Chernova, Sergey N. Kashtanov, Evgeny I. Rogaev

**Affiliations:** 1 Laboratory of Evolutionary genomics, Department of Genomics and Human Genetics, Vavilov Institute of General Genetics, Russian Academy of Sciences, 119333 Moscow, Russia; an.manakhov@gmail.com (A.D.M.); mingmary@mail.ru (M.Y.M.); an_tati@mail.ru (T.V.A.); 2 Center for Genetics and Genetic Technologies, Faculty of Biology, Lomonosov Moscow State University, 119192 Moscow, Russia; 3 Center for Genetics and Life Science, Sirius University of Science and Technology, 354340 Sochi, Russia; 4 Department of Animal Genetics, Vavilov Institute of General Genetics, Russian Academy of Sciences, 119333 Moscow, Russia; pffilimonov@yandex.ru (P.A.F.); stellarashes@yandex.ru (A.A.O.); snkashtanov@mail.ru (S.N.K.); 5 Puschkinsky State Fur Farm, Puschkinsky District, 141214 Moscow, Russia; achernova57@mail.ru; 6 Department of Psychiatry, University of Massachusetts Medical School, Worcester, MA 01604, USA

**Keywords:** sable, *Martes zibellina*, coat color, pastel, *TYRP1*

## Abstract

Sable (*Martes zibellina*) is one of the most valuable species of fur animals. Wild-type sable fur color varies from sandy-yellow to black. Farm breeding and 90 years of directional selection have resulted in a generation of several sable breeds with a completely black coat color. In 2005, an unusually chocolate (pastel) puppy was born in the Puschkinsky State Fur Farm (Russia). We established that the pastel phenotype was inherited as a Mendelian autosomal recessive trait. We performed whole-genome sequencing of the sables with pastel fur color and identified a frameshift variant in the gene encoding membrane-bound tyrosinase-like enzyme (*TYRP1*). TYRP1 is involved in the stability of the tyrosinase enzyme and participates in the synthesis of eumelanin. These data represent the first reported variant linked to fur color in sables and reveal the molecular genetic basis for pastel color pigmentation. These data are also useful for tracking economically valuable fur traits in sable breeding programs.

## 1. Introduction

**Figure 1:**
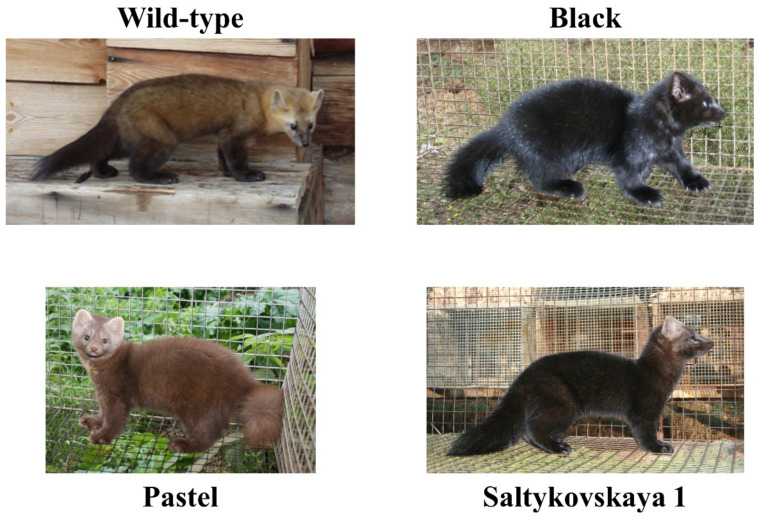
Sables with wild-type, black, Saltykovskaya 1 and pastel phenotypes.

Sable (*Martes zibellina*) has a wide distribution in Eurasia from the western slopes of the northern Urals to the Pacific coast and eastern Pacific islands. There are several geographical subtypes of *Martes zibellina* with a variety of morphological characteristics such as body weight and length and their fur color varies from sandy-yellow to black (Figure 1) [1,2,3]. Sable is a valuable animal species in the fur industry. Sable fur has been the most expensive fur on the market, with high demand worldwide, for several centuries [4].

At present, the most numerous wild sable populations exist in the European and Asian areas of the Russian Federation. By the beginning of the 20th century, due to commercial hunting, there was an overall decline in sable livestock and the elimination of several natural populations of sable in Russia. During that time, the first sable farms, where animals are maintained in cages, were created. The initial gene pool of sable farm populations in Russia was formed in the Puschkinsky State Fur Farm, Moscow region (established in 1929), by crossing approximately 1000 animals from natural populations in Siberia and the Far East [4,5].

The main aim of sable selection was to gradually darken the fur color by selection over multiple generations as dark pelts have been more valuable than those with wild-type coloration. Industrial farming and directional selection have resulted in two sable types that differ in fur pigmentation intensity: black sable and Saltykovskaya 1 (Figure 1) [6].

**Figure 2:**
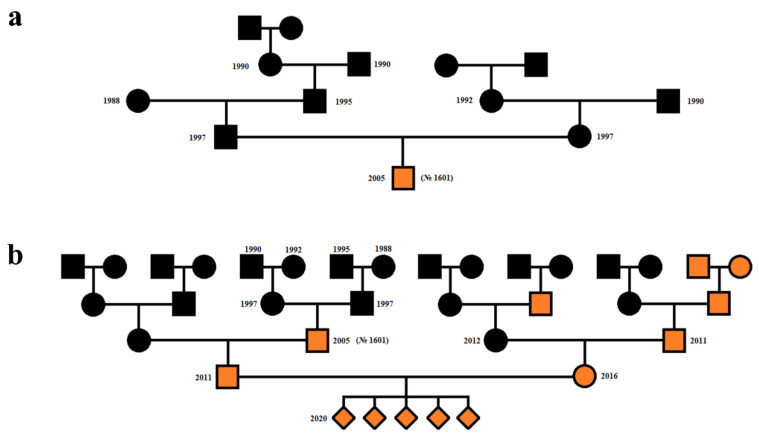
Pedigrees of pastel sables. (**a**). Genealogy of the first pastel sable (No. 1601), born in 2005. (**b**). The results of purebred pastel pairings. Squares indicate males, circles indicate females, a rhombus indicates unknown sex. Black indicates sables with a black coat, orange indicates pastel sables.

In 2005, after 90 years of farm breeding in the Puschkinsky State Fur Farm, an unusually brown pastel puppy (No.1601) was born to a pair of black sables (Figure 2a). This was the first sable fur color mutant registered in sable cage breeding.

Brown fur color is popular and valuable for another industrially farmed fur animal, the American mink (*Neovison vison*). At least twelve brown fur colors have been described for this animal [7]. We and others revealed links between several fur colors including brown colors and variants in certain genes in mink [8,9].

Here, we performed a genetic analysis of the inheritance of the pastel fur color trait in sables and searched for genetic factors that determined this phenotype by a whole-genome sequencing approach.

## 2. Materials and Methods

All methods were carried out in accordance with relevant guidelines and regulations for laboratory work. The Ethical Committee of the Vavilov Institute of General Genetics (Russian Academy of Sciences) reviewed and approved this study, including the whole-genome and target-gene sequencing studies of animals and the publication of the results (protocol code 5 and date of approval is 20 December 2016).

Samples (tongues) collected from farm-bred sables from the Puschkinsky State Fur Farm (Moscow region, Russia) were used in this study. No animals were killed specifically for the purposes of this study. A distinctive feature of sable farm breeding is the division of the population into several subpopulations that are maintained for long periods of time. The polygamy rate between males and females is 1:4. Samples from two subpopulations (**#**1, where pastel animals were bred—14 pastel; **#**2, where black animals were bred—13 black sables) were used. All samples were collected in 2019. The collected tissues were rapidly dissected, frozen in liquid nitrogen and stored at −70 °C until DNA extraction.

Additionally, we used sample collections of 44 wild sables from natural populations (Table S1). The populations were selected on the basis of the archive data analysis, which demonstrated that the Puschkinsky sable farm population was formed in the years between 1929 and 1947 by animals from the eastern part of the species’ range [4,5]. The biological samples were collected during the period from 2012 to 2019 in the area where sable hunting is legal. All samples from natural and farm populations are included in the collection deposited at the Vavilov Institute of General Genetics, Russian Academy of Sciences, Moscow. None of the animals were killed for the purpose of adding samples to the collection for this study.

Genomic DNA from sable tissues was extracted using QIAGEN Mini Spin Columns following the manufacturer’s protocol (QIAGEN, Hilden, Germany). Library preparations from two farm-bred pastels and one wild sable from Sakhalin were performed using a TruSeq PCR Free Kit (Illumina, San Diego, CA, USA) following the manufacturer’s protocol. A library validation was performed using an Agilent 2100 Bioanalyzer with a DNA High Sensitivity chip (Agilent, Lexington, MA, USA) and quantified via qPCR using a KAPA Library Quantification Illumina Kit protocol (KAPA Biosystems, Wilmington, MA, USA). Paired-end libraries were sequenced in 2 × 97 cycles using the Illumina TruSeq SBS v3 kit (Illumina, San Diego, CA, USA) on a HiSeq 2000/2500 sequencer (Illumina, San Diego, CA, USA) at the Vavilov Institute of General Genetics RAS (Moscow, Russia) and 2 × 151 cycles using the Illumina NovaSeq S4 kit (Illumina, San Diego, CA, USA) on a NovaSeq 6000 sequencer at the Genetico Company (Moscow, Russia). Datasets of whole-genome sequences of the three sables were deposited into the NCBI SRA database with the accession number PRJNA683151.

The Illumina paired-end sequencing data was filtered for low read quality and adaptor trimmed with AdapterRemoval v2 (--trimqualities --minquality 20 --minadapteroverlap 5 --minlength 50) [10].

The resulting reads were mapped to the ferret (*Mustela putorius furo*) genome (MusPutFur1.0) using a BWA-MEM algorithm (with default settings) [11]. Duplicate reads were detected using the MarkDuplicates algorithm from Picard tools v.1.133 [12] and excluded from further analyses.

Genetic variants in the sequenced sable genomes were predicted using the Genome Analysis Toolkit (GATK) HaplotypeCaller (with default arguments) package version 4.0 [13].

To detect the genetic factors underlying the pastel phenotype, we selected common homozygous variants (with a depth of coverage greater than 2 in for each sable) that were not homozygous in the standard dark brown wild-type sable from the Sakhalin population.

Annotation and effect predictions of selected variants were performed in SnpEff [14] with the ferret genome annotation (Ensemble MusPutFur1.0.99).

We performed Sanger sequencing to confirm the selected variants. Primers for PCR amplification were designed in Primer3 software (Table S2) and PCR was performed using the GenPack PCR Core (Isogen, Moscow, Russia). The resultant amplicons were cleaned using a Cleanup Standard Kit (Evrogen, Moscow, Russia) and processed using the BigDye Terminator v3.1 Cycle Sequencing Kit (Applied Biosystems, MA, USA) following the manufacturers’ protocols. Probes were purified using a DyeEx 2.0 Spin Kit (QIAGEN, Hilden, Germany) and sequenced in a 3730xl DNA Analyzer (Applied Biosystems, Beverly, MA, USA).

## 3. Results

### 3.1. Pastel Fur Color Is Inherited as a Mendelian Autosomal Recessive Trait

Pastel is a light brown sable coat color (Figure 1). The intensity of the pigmentation is equally distributed over the entire area of the sable pelt.

We performed a genetic hybrid analysis of pastel fur color inheritance in the subpopulation of sables from the Puschkinsky State Fur Farm. In total, 73 pairs of sables, which produced 237 puppies in 2019, were studied (Table 1).

In purebred pairings (i.e., both parents had the pastel fur trait), all puppies were born with pastel fur coats. The number of pairs was seven and the number of puppies was 21 (Figure 2b).

In crossbred pairings (27 pairs) of pastel and black sables, 22 pairs gave only black fur color offspring (71 puppies) and five pairs gave puppies of both fur colors (11 pastel and 11 with black fur color). Therefore, it was suggested that a few black sables were heterozygous for the pastel variant. The observed ratio of black and pastel animals among offspring for these five pairs was 1:1, which corresponds to a Mendelian inheritance.

In the offspring of the third group, purebred (both parents had black fur color, 37 pairs), 35 pairs gave only black fur color offspring (117 puppies) and two pairs gave offspring of both fur colors (four black and two pastel puppies). A detailed analysis indicated that the ratio was three black puppies to one pastel puppy in the offspring of the first pair and one black to one pastel puppy in the offspring of the second pair.

Taken together, our data suggest that sable pastel fur color is inherited as a Mendelian autosomal recessive trait.

### 3.2. Pastel Fur Color Is a Result of a Frameshift Variant in the TYRP1 Gene

We performed whole-genome sequencing to compare the genomes of two sable animals with pastel and one with wild-type fur color. The resulting genome coverages were 6x for pastel animals and 10 × for wild-type animal (Table S3).

**Figure 3:**
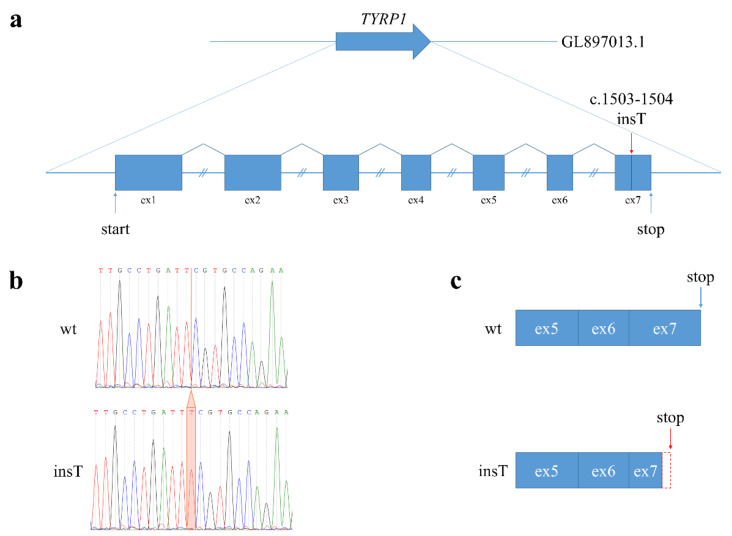
Effect of *TYRP1^b^* variant. (**a**). Structure of the sable *TYRP1* gene. The red line indicates the *TYRP1^b^* variant. Equal intron sizes are shown for simplification. (**b**). An electrophoretogram of the Sanger sequencing of the *TYRP1* gDNA exon 7. The orange box highlights a single nucleotide insertion in pastel sable homozygous for the *TYRP1^b^* variant. (**c**). The effect of the *TYRP1^b^* variant on the *TYRP1* transcript. This variant is predicted to result in a frameshift (indicated as a red dotted box) at the 502 position and create a premature stop codon at the 509 position (indicated as a red arrow).

We searched for homozygous variants across the genome that occurred in both analyzed pastel sables but not in the wild-type animal (Table S4). We then prioritized for further analysis those variations that were observed in protein encoding regions (gene exons) (Table S5). The genes bearing such variations were analyzed further for their direct or indirect potential involvement in the regulation of the pigmentation (Table S6). Ultimately, we found a homozygous single nucleotide insertion (GL897013.1: 5636116_5636117insA (*TYRP1*:c.1503_1504insT; TYRP1:p. Arg502SerfsTer8)), hereinafter referred to as *TYRP1^b^*, in the seventh exon of the tyrosinase-related protein 1 gene (*TYRP1*). This insertion is a potential loss-of-function variant of the TYRP1 protein (Figure 3).

We used Sanger sequencing and found that all tested pastel samples were homozygous for the *TYRP1^b^* variant. Eleven black farm-bred sables were homozygous for the reference allele and two black sables were heterozygotes for the *TYRP1^b^* variant. Additionally, we tested 44 wild sables from natural Russian populations and identified no *TYRP1^b^* variant, homozygous or heterozygous, in natural populations (Table 2).

Taken together, our data suggest that the *TYRP1^b^* variant has a causative link to the pastel fur color phenotype.

## 4. Discussion

The first genetic variant that affected fur color in sables was registered in 2005 in the Puschkinsky State Fur Farm population. The puppy with a pastel coat color was the offspring of a pair of black sables (Figure 2a). Previously, these parents had nine black puppies in three crossings. A genealogical analysis of the pedigree of the first pastel sable parents showed that all of their ancestors for three generations had a black fur color.

Our analysis of hybrid crosses performed in 2019–2020 confirmed that a pastel coat color is inherited as a Mendelian autosomal recessive trait (Table 1). A high population size is a necessary factor for the manifestation of phenotypes caused by recessive mutations. In farm breeding of American mink, the number of animals produced reached 15–20 thousand puppies annually in just 30 years from the beginning of farm breeding. Since that time, several farms have reported many mutant fur color phenotypes in American mink [7,15]. The number of sable offspring reached 15 thousand puppies annually only now after 85 years of farm breeding because sables have a lower fertility and later puberty than American mink. Thus, we recorded here the first recessive mutant fur color phenotype in sables.

The present study described the genetic variant in the *TYRP1* gene that resulted in the pastel coat color phenotype in sables. It is the first mapped color-related gene in sables. The *TYRP1* gene encodes a membrane-bound tyrosinase-like enzyme that is expressed in both melanocytes and the retinal epithelium. In tandem with dopachrome tautomerase (DCT), the TYRP1 enzyme catalyzes the oxidation of intermediates in the synthesis of eumelanin and is involved in the stability of the tyrosinase enzyme [16,17].

Mutations in the *TYRP1* gene have been described in several domestic animals with inherited brown coat colors including mice [18], cats (OMIA 001249-9685), dogs (OMIA 001249-9615), rabbits (OMIA 001249-9986), cattle (OMIA 001249-9913), pigs (OMIA 001249-9823), goats (OMIA 001249-9925), sheep (OMIA 001249-9940) and American mink (OMIA 001249-452646). In humans, mutations in the *TYRP1* gene are known to cause oculocutaneous albinism type 3 (OMIM 203290). 

The *TYRP1^b^* variant was predicted to result in a frameshift at amino acid position 502 of the protein, leading to the origin of a premature stop codon at the 509 position. Truncated mutant proteins completely lose the flexible C-terminal cytoplasmic domain (amino acids 502–537), which seems to be involved in targeting TYRP1 to the melanosomal membrane [19,20]. Previously, a missense mutation in the cytoplasmic domain of the TYRP1 protein (TYPR1:p. Pro513Agr) was identified in a patient with oculocutaneous albinism type 3 [21]. We hypothesized that the disturbance of TYRP1 trafficking to melanosomes may lead to a decrease in melanin production that results in the light brown fur color.

We suggest that the identified variant in *TYRP1* is causative for the pastel sable phenotype in the Puschkinsky State Fur Farm sable population. According to archive data, the Puschkinsky State Fur Farm population was formed from 1000 sables from natural populations of several regions in Siberia and the Far East [4,5]. To test the hypothesis that the pastel variant may be present in wild sable populations, we performed genotyping of animals from several natural populations (Table S1) for the *TYPR1^b^* variant. We did not identify any homozygous or heterozygous *TYRP1^b^* animals in natural populations (Table 2). Therefore, we hypothesize that the sable pastel variant originated de novo in the Puschkinsky State Fur Farm.

## 5. Conclusions

In conclusion, we presented a pilot study describing the identification of the first mutant gene for the fur color phenotype in sables. The data contribute to the understanding of the molecular genetic mechanisms underlying intraspecies phenotype diversity. The data may also be useful for selective breeding programs in the fur industry.

## Figures and Tables

**Table 1:** Results of black and pastel sables’ crosses.

Crosses		Offspring				
	Litter	Offspring Number	Black	Pastel	χ^2^	*p*
Pastel × pastel	7	21	0	21	-	-
Black × pastel	22	71	71	0	-	-
	5	22	11	11	0.00	1.00
Black × black	35	117	117	0	-	-
	2	6	4	2	0.22	0.64
Total	73	237	203	34	-	-

**Table 2:** Results of *TYRP1^b^* genotyping in sables.

Phenotype Name	Population	Genotypes for the *TYRP1^b^* Alleles *			
		wt/wt	wt/insT	insT/insT	∑
Pastel	Farm-bred	0	0	14	14
Black		11	2	0	13
Wild-type	Natural population	44	0	0	44

* wt is wild-type allele; insT is allele with insertion.

## Data Availability

The datasets generated during the current study were deposited into the NCBI SRA database and can be accessed with the BioProject accession number PRJNA683151 (https://www.ncbi.nlm.nih.gov/sra/PRJNA683151).

## Supplementary Materials

The following are available online at https://www.mdpi.com/2073-4425/12/2/157/s1, Table S1: Collection of sables from natural population; Table S2: Primer sequences used for gDNA amplification; Table S3: Results of the sequencing of sable genomes. Statistics were calculated using Samtools and Picard software. The ferret (*Mustela putorius furo*) genome (MusPutFur1.0) was used as a reference. Table S4. List of homozygous genetic variants occurring in both analyzed pastel sables but not in wild-type animals. Table S5. List of homozygous genetic variants occurring in both analyzed pastel sables but not in wild-type animals that were observed in protein encoding regions. Table S6. List of homozygous genetic variants occurring in both analyzed pastel sables but not in wild-type animals that were observed in protein encoding regions of genes that involved in the regulation of pigmentation.
