# Supplementary material for: Genome Analysis of Sable Fur Color Links a Lightened Pigmentation Phenotype to a Frameshift Variant in the Tyrosinase-Related Protein 1 Gene
Source: Genes (Basel). 2021 Jan 25;12(2):157. doi: 10.3390/genes12020157 (PMC7911072; doi:10.3390/genes12020157)
Supplement: Supplementary file 1 [file genes-12-00157-s001.zip › Sable_TYRP1_SM_v2.pdf]

Genome analysis of sable fur colour links lightened pigmentation phenotype to a frameshift variant in the tyrosinase-related protein 1 gene

Andrey D. Manakhov [1, 2, 3] (orcid: 0000-0002-5163-8747), Maria Yu. Mintseva [1] (orcid: 0000-0003-4613-471X), Tatiana V. Andreeva [1, 2], Pavel A. Filimonov [4], Alexey A. Onokhov [4], Irina E. Chernova [5], Sergey N. Kashtanov [4], Evgeny I. Rogaev [1, 2, 3, 6] *

[1] Department of Genomics and Human Genetics, Laboratory of Evolutionary genomics, Vavilov Institute of General Genetics, Russian Academy of Sciences, Moscow, 119333, Russia

[2] Center for Genetics and Genetic Technologies, Faculty of Biology, Lomonosov Moscow State University, Moscow, 119192, Russia

[3] Sirius University of Science and Technology, Sochi, 354340, Russia

[4] Department of Animal Genetics, Vavilov Institute of General Genetics, Russian Academy of Sciences, Moscow, 119333, Russia

[5] Puschkinsky State Fur Farm, Puschkinsky district, Moscow region, 141214, Russia

[6] Department of Psychiatry, University of Massachusetts Medical School, Worcester, MA 01604, USA

* Corresponding author (rogaev@vigg.ru; Vavilov Institute of General Genetics, Russian Academy of Sciences, Moscow, 119333, Russia; tel: 8 (499) 135-50-61)

Supplementary

Table S1. Collection of sables from natural population.

**Areal part**	**Population**	**Coordinates**	**Number of samples**	**∑**
West	Northern Ural	N61.50°, E56.50°	2	5
	Omsk	N57.29°, E71.32°	2	
	Yenisei	N62.29°, E86.16°	1	
Central	Altai	N50.93°, E84.76°	2	7
	Tunguska	N61.69°, E89.86°	1	
	Baikal	N52.41°, E100.20°	3	
	Tura	N64.28°, E100.21°	1	
East	Sikhote-Alin	N49.44°, E136.55°	10	32
	Sakhalin	N46.67°, E141.85°	9	
	Iturup	N45.25°, E147.88°	2	
	Kamchatka	N57.76°, E158.67°	11	

Table S2. Primer sequences used for gDNA amplification.

Primer name	Primer sequence	Expected amplicon size (bp)	Annealing temp (°C)
gDNA TYRP1 ex 7 F	TGGTTCAAACCCTACAGCAT	588	58
gDNA TYRP1 ex 7 R	ACAAGTGCATGCATAAGGAAG		

Table S3. Results of sequencing of sable genomes. Statistics were calculated using Samtools [1] and Picard software. The ferret (*Mustela putorius furo*) genome (MusPutFur1.0) was used as a reference.

Sample	Population	Colour	Mapped %	Duplicates %	Coverage
sable_2506	Sakhalin	Wild type	95.89	3.07	9.96
sable_8056	Farm-bred	Pastel	92.50	1.23	5.81
sable_8078	Farm-bred	Pastel	92.70	1.29	5.61

1. Li H, Handsaker B, Wysoker A, Fennell T, Ruan J, Homer N, et al. The Sequence Alignment/Map format and SAMtools. Bioinformatics. 2009;25:2078–9.

Tables S4. List of homozygous genetic variants occurred in both analysed pastel sables but not in wild-type animal.

Tables S5. List of homozygous genetic variants occurred in both analysed pastel sables but not in wild-type animal that were observed in protein-encoding regions.

Tables S6. List of homozygous genetic variants occurred in both analysed pastel sables but not in wild-type animal that were observed in protein-encoding regions of genes that involved in the regulation of pigmentation.
